# A Case of DeSanto-Shinawi Syndrome in Bahrain with a Novel Mutation

**DOI:** 10.1155/2020/8820966

**Published:** 2020-10-16

**Authors:** Zahra Alsahlawi, Mohamed Jailani, Husain Alaradi, Abdulaziz AlAbbad

**Affiliations:** ^1^Department of Pediatrics, Salmaniya Medical Complex, Manama, Bahrain; ^2^Salmaniya Medical Complex, Manama, Bahrain

## Abstract

DeSanto-Shinawi syndrome is a rare genetic condition caused by loss-of-function mutation in *WAC.* It is characterized by dysmorphic features, intellectual disability, and behavioral abnormalities. In this case report, we describe the clinical features and genotype of a patient with a novel mutation 1346C > A in *WAC*. This patient's dysmorphic features include a prominent forehead, bulbous nasal tip, macroglossia, deep-set eyes, and malar hypoplasia. This patient also showed signs of intellectual disability and behavioral abnormalities such as night terrors. These findings are consistent with those described in earlier reports. Here, we report new findings of epilepsy and recurrent skin infections which had not been reported in prior studies.

## 1. Background

DeSanto-Shinawi syndrome (DESSH, OMIM 616708) is an autosomal dominant genetic condition caused by loss-of-function mutations in *WAC* and is characterized by a multitude of dysmorphic features such as a broad forehead, bulbous nasal tip, posteriorly rotated ears, deep-set eyes, brachycephaly along with behavioral abnormalities, and intellectual disability [1–4]. Twenty cases have been reported with DESSH, and all have been identified to have a de novo mutation in the *WAC* gene [4–7]. Four additional individuals with a *WAC* de novo mutation have also been reported but do not have the full spectrum of physical findings as the previously reported twenty cases [8, 9]. One of which has been reported to have a somatic mosaicism in *WAC* [9]. This brings the total to twenty-four patients, twenty of whom have the full spectrum and four do not have the full spectrum.

*WAC*, the gene responsible for this condition, is mapped to the region 10p12.1. The protein product of this gene is a WW domain-containing adapter protein that plays a vital role as a transcription regulator in several biological processes. These biological processes include autophagy, Golgi reformation, and cell-cycle checkpoints [10–14].

Here, we report a novel mutation in a 38-month-old patient who presented to Salmaniya Medical Complex in Bahrain with DESSH. The full phenotypic spectrum of this condition is yet to be defined, so comparing the findings seen in our patient with findings in earlier reported cases with mutations in *WAC* will further delineate the phenotypic and genotypic spectrum of this condition.

## 2. Case Presentation

The proband is a 38-month-old male who presented to Salmaniya Medical Complex in the Kingdom of Bahrain. He was born at 39 weeks of gestation to second-degree once removed consanguineous parents of Syrian origins. The father (38 years old) and the mother (35 years old) are not known to have any genetic or chronic diseases. The parents have three daughters aged 11, 10, and 6 years, all of whom are healthy. The proband is a product of a normal vaginal delivery. There were no complications during pregnancy, nor during delivery. He was born with Apgar scores of 9 at 1 min and 10 at 5 min. His birth weight was 3.05 kg (6^th^ percentile), length was 53 cm (54^th^ percentile), and head circumference was 34 cm (6^th^ percentile).

He was admitted four times for lower respiratory tract infections: twice for pneumonia, once for bronchiolitis, and once for bronchitis. He also had a total of ten clinically documented upper respiratory tract infections that resolved spontaneously at home. They were associated with otitis media and conjunctivitis. The patient was treated once for a cutaneous abscess with antibiotics. He was also treated for a skin rash, impetigo, and candidal stomatitis. These infections were documented clinically, without microbiological or radiological documentations. The proband had two clinically documented generalized tonic-clonic seizures at one and three months of age and one undocumented generalized tonic-clonic seizure at seven months of age. He was investigated with an electroencephalograph (EEG) at seven months of age due to repeated seizures. The EEG showed no abnormalities. It was performed when the patient was not on antiepileptic drugs. There was no special reference to status epilepticus during sleep when the EEG was performed.

The proband's developmental delay was noticed at one year of age. By the age of 36 months, he could only say his name and a few two-word sentences. His gross motor development was also delayed, where he started crawling by 15 months, walking by 26 months, running by 30 months, and walking up the stairs by 36 months of age. The proband's fine motor skill development was also delayed, where he developed a pincer grasp by 24 months of age. With regard to his social skills, he started to wave by 32 months of age.

The proband was breastfed and given formula milk up to the age of three months and then was switched to formula feeding up to the age of 26 months. At the age of 26 months, he started having a regular diet. The proband currently has a normal diet, with no known food allergies, and has never exhibited feeding problems. The proband had passed stool once or twice a week until the age of 24 months, and since then, he has been having bowel movements once or twice per day.

The proband displayed behavioral abnormalities. He had displayed daily night terrors that stopped occurring gradually by the age of 24 months. Hyperactivity was noted by the proband's family members, and that was not found to be clinically significant by a psychiatric assessment.

**Figure 1:**
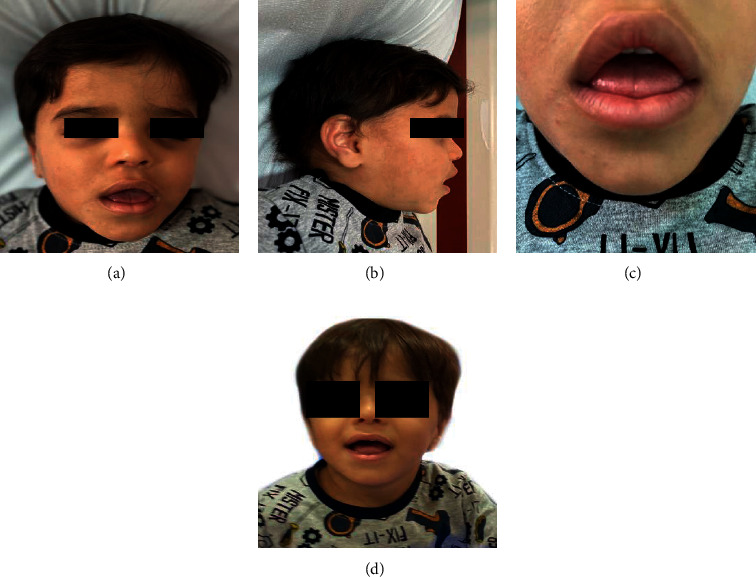
Patient's face and tongue showing facial dysmorphic features.

**Figure 2:**
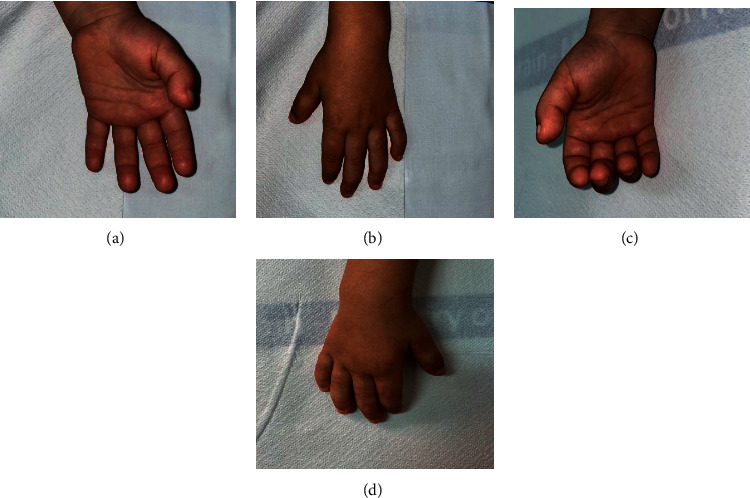
Patient's hands showing short fingers.

On physical examination at 32 months of age, his height was 95 cm (10^th^ percentile) and his weight was 14.0 kg (50^th^ percentile). The proband's dysmorphic features are as follows: a prominent forehead, posteriorly rotated ears, low nasal bridge, bulbous nasal tip, malar hypoplasia, deep-set eyes, macroglossia, prominent upper lip, flat philtrum (Figure 1), and short fingers (Figure 2).

**Figure 3:**
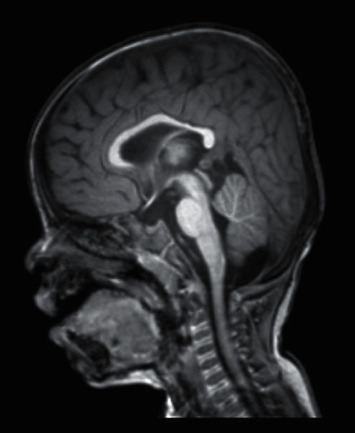
Brain MRI showing normal and progressive myelination but mild loss of volume in the corpus callosum.

A brain multiplanar multisequenced magnetic resonance imaging (MRI) was performed at 11 months of age and showed normal and progressive myelination but mild loss of volume in the corpus callosum (Figure 3). A repeat MRI to evaluate the patient after the second year of life when myelination is more complete was not performed.

He was also evaluated for recurrent infections at 36 months of age, and lab investigations showed the following: complete blood count, liver function tests, renal function tests, and a complete metabolic panel were within normal limits; immunoglobulin levels were as follows: immunoglobulin A, 1.28 g/L (normal 0.30–1.3 g/L); immunoglobulin G, 10.3 g/L (normal 3.1–15.8 g/L); and immunoglobulin M, 1.14 g/L (normal 0.50–2.20).

The proband had an ENT evaluation at 33 months of age which did not reveal any abnormalities. He was initially evaluated for Beckwith-Wiedemann syndrome given his prominent macroglossia; accordingly, a methylation and deletion/duplication analysis of 11p15.5 was carried out. The test revealed normal methylation patterns of DMR1 (H19), and the DMR2 (KCNQ1OT1) imprinting regions were observed. There were no deletions or duplications in the 11p15 region.

## 3. Genetic Analysis

**Figure 4:**
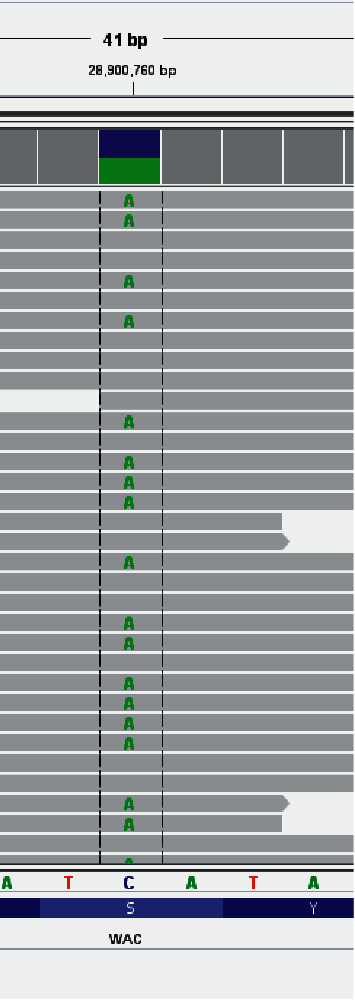
*WAC* mutation analysis showing 1346C > A.

We performed whole exome sequencing (WES) on DNA from peripheral blood of the patient and his parents. Genetic analysis was carried out by Centogene laboratory in Germany. Variants found in the patient were compared with variants found in the parents. Findings were then confirmed using Sanger sequencing. WES revealed a heterozygous novel mutation in *WAC* (NM_016628.4 : c.1346 C > A) p.(Ser449∗) (Figure 4). Furthermore, known mutations under the epilepsy and hypotonia panels were not found. Both parents tested negative for this mutation. Accordingly, this was confirmed as a de novo mutation. This mutation creates a premature stop codon in exon 10. This mutation was confirmed by Sanger sequencing.

## 4. Discussion

Here, we report a case of DeSanto-Shinawi syndrome caused by a novel heterozygous mutation (NM_016628.4 : c.1346C > A) p.(Ser449∗) in *WAC*. The phenotype of this patient was very similar to the phenotype of patients reported in earlier cases. The patient's most important clinical features were as follows: prominent forehead, low nasal bridge, bulbous nasal tip, malar hypoplasia, deep-set eyes, macroglossia, flat philtrum (Table 1), night terrors, intellectual disability, epilepsy, and recurrent respiratory infections. Epilepsy was seen in five previous patients, two of which had developed encephalopathy related to status epilepticus during sleep (ESES). Recurrent skin infections are a new finding that had not been previously reported.

WAC, the protein product of the gene involved in this condition, plays a vital role in gene transcription, microtubule development, autophagy, and Golgi apparatus function [6, 11]. In addition to these functions, WAC also plays a role in pathogen recognition and antigen presentation. Accordingly, an interesting hypothesis was put forth by Vanegas et al. regarding the association of *WAC* haploinsufficiency, recurrent infections, and hypogammaglobulinemia seen in the case reported by them [4]. The patient we report was treated for multiple respiratory and skin infections, and this warranted an immunological workup, but the results turned out to be normal.

Epilepsy was seen in one of the patients reported by Lungtenberg, but that patient also carried a de novo *MIB1* mutation. That patient had the more severe phenotype, with severe intellectual disability, epilepsy, and absence of speech. Lungtenberg hypothesized that the mutation in *MIB1* is unlikely to be a potential modifier and that this patient might be on the severe end of the phenotypic spectrum caused by *WAC* haploinsufficiency [6]. The patient we report does not carry another mutation but has had three episodes of seizures. This is in line with the hypothesis put forth by Lungtenberg. Epilepsy was also reported by Leonardi, in which the patient developed ESES. This was also reported by Zhang in a paper published only in Chinese [8].

ESES is a rare epileptic syndrome, with an onset during childhood. It presents with epilepsy, cognitive regression, and epileptiform activity during nonrapid eye movement sleep. The EEG pattern observed is of a near-continuous diffuse or bilateral spike-wave discharges (1.5–3 Hz) [15]. The reason behind this phenomenon is unknown, but it has been postulated that these findings are due to abnormal hyperactivation of the thalamic oscillatory circuit, which is an interaction between the inhibitory GABAergic neurons and the excitatory glutaminergic neurons. Accordingly, such sleep epileptiform discharges may interrupt cortical information processing centers which can lead to intellectual disability [16, 17].

The EEG performed on this patient showed no abnormalities. An MRI however showed a mild loss of volume in the corpus callosum. This finding has not been reported in any of the previous cases. Since the corpus callosum is made of white matter and has no firing neurons, it cannot act as an epileptic focus [18]. Epilepsy in this patient might be an isolated finding, or an association with the more severe phenotype of *WAC* haploinsufficiency.

## 5. Conclusion

The data presented in this paper further delineate the phenotypic and genotypic spectrum of DeSanto-Shinawi syndrome. The presence of epilepsy in this patient suggests that *WAC* should be included in the genetic screening of epilepsy as suggested by Leonardi [9]. Furthermore, ESES or epilepsy along with the characteristic dysmorphic features could be highly suggestive of DESSH.

## Figures and Tables

**Table 1:** Findings in comparison with previously reported *WAC* cases in the literature. The dysmorphic features reported in Zhang et al., 2019 [8] is presented in this table according to the clinical illustration found in https://www.sohu.com/a/354042751_387855. CC, cranial circumference; N.R., not reported; +, present; −, absent.

	DeSanto et al., 2015	Lugtenberg et al., 2016	Uehara et al., 2018	Vanegas et al., 2018	Zhang et al., 2019	Leonardi et al., 2020	This Report
Sex	5 F, 1 M	6 F, 4 M	3 M	M	F	1 F, 2 M	M
Age	1.3–11 y/o	1.5–22 y/o	3–22 y/o	4 y/o	5.8 y/o	7–17 y/o	3.2 y/o
Normal Perinatal Period	2/6	6/10	3/3	+	+	2/3	+
Delayed Physical Growth	2/6	3/10	2/3	+	−	2/3	+
CC Size Anomaly	N.R.	2/10	1/3	+	−	2/3	+

*Development*							
Intellectual Disability	3/6	8/10	3/3	+	+	2/3	+
Language Delay	6/6	9/10	3/3	+	+	3/3	+
Motor Delay	6/6	9/10	3/3	+	+	3/3	+

*Behavioral Problems*							
Autistic Features	1/6	4/9	0/3	−	N.R.	2/3	−
Hyperactivity	3/6	4/10	0/3	+	N.R.	1/3	−
Anxiety	3/6	3/10	1/3	+	N.R.	1/3	−
Sleep Disturbance	2/6	6/10	0/3	-	+	2/3	+
Stereotypies	1/6	N.R.	0/3	-	N.R.	2/3	−

*Neurological*							
Hypotonia	6/6	7/9	0/3	+	+	2/3	+
Seizures	2/6	1/9	0/3	−	+ (focal)	1/3	+
Epilepsy	1/6	1/9	0/3	−	+	1/3 (focal)	+

*Ocular*							
Vision	2/6	5/10	1/3	−	N.R.	1/3	−
Strabismus	3/6	3/10	1/3	−	N.R.	1/3	−

*Dysmorphisms*							
Facial Shape Dysmorphology	N.R.	5/10	2/3	−	+	1/3	+
Prominent Forehead	6/6	10/10	1/3	+	+	1/3	+
Bulbous Nasal Tip	5/6	N.R.	3/3	+	+	2/3	+
Long or Downslanting Palpebral Fissures	1/6	5/10	3/3	−	−	1/3	+
Synophrys	3/6	2/10	1/3	+	+	1/3	-
Deep Set Eyes	2/6	5/10	0/3	+	+	2/3	+
Full Lips–Think Upper Lip	3/6	N.R.	1/3	-	−	0/3	+
Low-set Ears	3/6	N.R.	1/3	+	−	0/3	−
Hirsutism/Hypertricosis	2/6	1/10	1/3	+	−	1/3	+
Digital Anomalies	1/6	7/7 (3 N.R.)	3/3	+	N.R	1/3	+
Posteriorly Rotated Ears	3/6	10/10	N.R.	+	−	N.R.	+
Preauricular Pit	1/6	N.R.	1/3	−	−	N.R.	−
Low Nasal Bridge	6/6	N.R.	N.R.	+	+	N.R.	+
Malar Hypoplasia	1/6	N.R.	N.R.	+	+	N.R.	+
Hypertelorism	2/6	10/10	N.R.	+	+	N.R.	−
Macroglossia	N.R.	10/10	N.R.	+	N.R.	N.R.	+

*Other*							
Feeding Difficulties	4/6	4/10	0/3	+	N.R.	1/3	−
Constipation	5/6	N.R.	1/3	−	N.R.	1/3	+
Frequent Infections	N.R.	6/8 (2 N.R.)	0/3	+	N.R.	0/3	+
Respiratory Infections	N.R.	6/8 (2 N.R.)	N.R.	+	N.R.	0/3	+
Skin Infections	N.R.	N.R.	N.R.	N.R.	N.R.	0/3	+
Hypogammaglobulinemia	N.R.	N.R.	N.R.	+	N.R.	N.R.	−
Hearing Abnormalities	2/6	0/2 (8 N.R.)	0/3	+	N.R.	0/3	−
EEG Abnormalities	0/2	0/1 (9 N.R.)	N.R.	N.R.	+	1/3	−
MRI Abnormalities	1/6	4/9	N.R.	−	−	0/3	+

## Data Availability

The data used to support the findings of this study are available from the corresponding author upon request.

## Abbreviations

DESSH: DeSanto-Shinawi syndrome
OMIM: Online Mendelian Inheritance in Man
EEG: Electroencephalograph
MRI: Magnetic resonance imaging
ESES: Electrical status epilepticus during sleep.
